# PRIMARY SPHINCTEROPLASTY COMPARING TWO DIFFERENT STITCHES IN ANAL FISTULA TREATMENT: EXPERIMENTAL STUDY IN RATS

**DOI:** 10.1590/0102-672020190001e1459

**Published:** 2019-12-09

**Authors:** Otávio Augusto Vendas TANUS, Carlos Henrique Marques dos SANTOS, Doroty Mesquita DOURADO, Andrea Lima CONDE, Fernanda GIUNCANSE, Isadora Ferreira de SOUZA, Izabela Oliveira COSTA, Rochelle Leite COSTA

**Affiliations:** 1 General Surgery Department, Universitary Hospital Maria Aparecida Pedrossian, Federal University of Mato Grosso do Sul; Campo Grande, MS, Brazil; 2 Anhanguera-Uniderp University, Campo Grande, MS, Brazil.

**Keywords:** Anal canal, Rectal fistula, Rats, Canal anal, Fístula retal, Ratos

## Abstract

**Background:**

Anal fistula is by definition the communication between the rectum or anal canal with the perineal region, which may be epithelialized and has cryptoglandular origin in 90% of cases. There are a large number of techniques for successfully treating trans-sphincteric fistulas of 20-50%, including primary sphincteroplasty, but it is not clear whether the material used would influence the outcome.

**Aim:**

To analyze the efficacy of polydioxanone and polypropylene wire in primary post-fistulotomy sphincteroplasty in the treatment of trans-sphincteric fistulas in rats.

**Methods:**

Thirty Wistar rats had transfixation of the anal sphincter with steel wire, which remained for 30 days for the development of the anal fistula. After this period, it was removed and four groups were formed: A (control) without treatment; B (fistulotomy) submitted to such procedure and curettage only; C (polidioxanone) in which sphincteroplasty was performed after fistulotomy with polydioxanone wire; D (polypropylene) submitted to the same treatment as group C, but with polypropylene wire. After 30 days, euthanasia and removal of the specimens were performed for qualitative histopathological analysis, measurement of the area between the muscular cables edges and evaluation of the degree of local fibrosis.

**Results:**

There was persistence of the anal fistula in all animals of group A. There were no significant differences between groups B, C and D regarding the distance of the muscular cables (p=0.078) and the degree of fibrosis caused by the different treatments (p=0.373).

**Conclusions:**

There was no difference between polydioxanone and polypropylene wires in post-fistulotomy primary sphincteroplasty, and this technique was not superior to simple fistulotomy in relation to the distance of the muscular cables nor did it present differences in relation to the degree of local fibrosis.

## RESUMO

**Racional:**

Fístula anal tem por definição a comunicação entre o reto ou canal anal com a região perineal, trajeto este que pode encontrar-se epitelizado e tem origem criptoglandular em 90% dos casos. Há grande número de técnicas para o tratamento das fístulas transesfincterianas com sucesso de 20-85%, incluindo-se a esfincteroplastia primária, mas não está claro se o material utilizado teria influência no resultado.

**Objetivo:**

Analisar a eficácia dos fios de polidioxanona e polipropileno na esfincteroplastia primária pós-fistulotomia no tratamento de fístulas transesfincterianas em ratos.

**Métodos:**

Utilizou-se 30 ratos Wistar com transfixação do esfíncter anal com fio de aço, que permaneceu por 30 dias para desenvolvimento da fístula anal. Após esse período, este fio foi removido e foram formados quatro grupos: A (controle) sem tratamento; B (fistulotomia) submetido a tal procedimento e curetagem apenas; C (polidioxanona) em que foi feita esfincteroplastia com fio polidioxanona após a fistulotomia; D (polipropileno) submetido ao mesmo tratamento do grupo C, porém com fio de polipropileno. Após 30 dias foi praticada a eutanásia e remoção dos espécimes, para análise histopatológica qualitativa, medição da área de afastamento dos cabos musculares e avaliação do grau de fibrose local.

**Resultados:**

Houve persistência da fístula em todos os animais do grupo A. Não houve diferença significativa entre os grupos B, C e D quanto ao afastamento dos cabos musculares (p=0,078) e ao grau de fibrose causado pelos diferentes tratamentos (p=0,373).

**Conclusões:**

Não houve diferença entre os fios de polidioxanona e polipropileno na esfincteroplastia primária pós-fistulotomia, e esta técnica não foi superior à simples fistulotomia quanto ao afastamento dos cabos musculares nem apresentou diferenças em relação ao grau de fibrose local.

## INTRODUCTION

Trans-sphincteric anal fistulae involving more than 30% of the external sphincter, although they may be treated by various techniques, have a proportionally higher cure rate as the risk of fecal incontinence increases^1^ ^,^ ^13^ ^,^ ^14^. The evaluation and classification in clinical practice can be done by magnetic resonance or endoanal ultrasound^10^ ^,^ ^15^. 

Among the therapeutic possibilities are the injection of fibrin glue, collagen plug, mucosal flap and ligation of intersphincteric fistulous tract (LIFT), techniques that have been used with rates of recurrence between 0-65% and incontinence post-procedure of 0-63%^7^. 

Among the therapeutic possibilities there is also the fistulotomy followed by primary sphincteroplasty. The primary suture of the anal sphincter after fistulotomy was initially used by Chassaignac in 1856 and Stephen and Smith in 1879, a technique that was abandoned due to the concern with surgical site infection^18^. 

Initially only the apposition of the extremities of the margins of the damaged anal sphincter was reported; however, the operation became popular after publication of Parks et al.^19^. This technique has been questioned because of its long-term success rate, which, like many other operations for functional diseases of the gastrointestinal tract, decreases over time. 

In the 1980s, new results with immediate sphincteroplasty after fistulotomy were published, reviving the idea that it is a potentially curative procedure and prevents anal deformity. After these studies, the method was used again in the treatment of complex fistulae^18^.

Currently, fistulotomy followed by immediate sphincteroplasty has been suggested as a therapeutic option in reducing postoperative fecal incontinence, with recurrence rates of less than 10% in some studies and postoperative incontinence ranging from 3.6% to 31.7%^6^. Considering this large variation in the results, we question the reason for such discrepancy, such as the complexity of the fistula, amount of sectioned sphincter, previous state of the sphincter tone, etc. The hypothesis presented here is that the material used for suturing may also have some influence, which is not yet analyzed in the published literature. 

Sphincteroplasty is generally indicated for patients with some obstetric damage to the anal sphincter during normal delivery after iatrogenic injury to cure anal abscess or complex anal fistula. It is unclear whether the cause of sphincter injury may affect the outcome, but one of the few studies addressing this subject suggests that patients with surgical trauma have more significant improvement than those with obstetric trauma^17^. Perhaps this difference is due to the fact that woman is exposed to deterioration of muscular trophism and innervation over the years, particularly after menopause, due to the drop in estrogen levels^4^.

Another detail regarding the operation includes the use of absorbable or nonabsorbable yarns. Old studies in sphincteroplasty were performed using catgut to approximate the sphincter margins, and the use of this material is determinant when analyzing the results in the long term. Catgut is produced from collagen derived from mammalian biological material, known to cause inflammatory response in tissues due to its being metabolized by proteolytic enzymes and phagocytosis. It is an unstable and unpredictable material. The literature suggests that the use of polydioxanone or polypropylene is better than the use of polyglactin or polyglycolic acid, since they require a longer period of time to be absorbed^9^. 

Due to these implications, we have chosen to evaluate the use of polydioxanone and polypropylene. Both are synthetic and monofilament yarns; however, the polydioxanone yarn is absorbable by hydrolysis, with an absorption time of approximately 90-180 days, causing small inflammatory reaction when compared to other suture materials such as catgut and cotton. Polypropylene is inabsorbable and biologically inert, even in the presence of infection, with high chemical resistance to acids, alkalis and enzymes^12^.

The aim of this study was to analyze the efficacy of polydioxanone and polypropylene yarns in primary post-fistulotomy sphincteroplasty in the treatment of trans-sphincteric fistulae in rats.

## METHODS

This study was approved by the Committee on Ethics in Animal Use (CEUA) of Federal University of Mato Grosso do Sul, under protocol no. 869/2017. 

Wistar rats (*Rattus norvegicus*, var. Albinus), male, adults, weighing approximately 300 g each, were studied. The animals were kept in a light/dark cycle (12/12 h), with controlled temperature at 22±1^o^ C and free access to water and food.

The rats were anesthetized for the fistulas creation by intraperitoneal administration of ketamine and xylazine, associated in the same syringe. The solution was composed for 1 ml of 10% ketamine and 1 ml of 2% xylazine, being for each 100 g of body weight, 0.1 ml of the anesthetic solution. 

**FIGURE 1:**
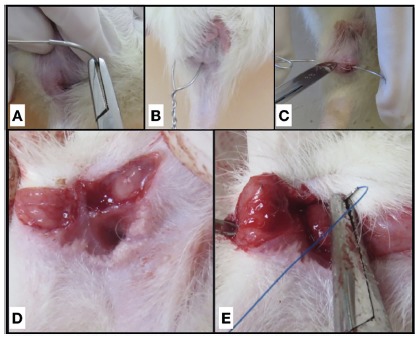
A) Steel wire traversing anal sphincter in the pectine line; B) twisted steel wire after placement through the anal sphincter; C) fistulotomy; D) sectioned sphincter after fistulotomy; E) primary sphincteroplasty after fistulotomy

After the anesthesia, the anal fistulas were made. The entire procedure was performed after antisepsis and asepsis care of the operated region. In the supine position, with the four limbs in abduction, the introduction of the needle of steel wire number five (Aciflex^®^) in the pectin line was placed in the lateral right position, crossing the anal sphincter and exiting in the perianal skin 1 cm from the anal margin (Figure 1A).

After that, the steel wire was cut and then made a loose knot through rotational movements^13^ (Figure 1B). 

After 30 days, the rats were distributed into four groups: control group (A n=5) subjected to the withdrawal of the steel wire, without further therapeutic actions; fistulotomy group (B, n=5) had the steel wire removed and immediately subjected to fistulotomy and curettage, the wound left for healing by second intention; polydioxanone group (C, n=10) subjected to yarn removal followed by immediately fistulotomy, curettage and primary sphincteroplasty with 4-0 polydioxanone thread; polypropylene group (D, n=10) had the steel wire removed and immediately subjected to fistulotomy, curettage and primary sphincteroplasty with 4-0 polypropylene wire.

The complete fistulotomy (Figure 1C) was performed under aseptic conditions with a cold blade scaler number 22, using the steel wire as a guide, incising the skin and the anal sphincter along the entire fistulous tract. After compressive hemostasis with sterile gauze for about 30 s, the fistulous tract was subjected to curettage until complete resection of all granulation tissue (Figure 1D), followed by new haemostasis. The terminoterminal sphincteroplasty (Figure 1E) was made with “U” stitches, with the respective threads of each group, keeping the knot facing the internal region. The skin was left open for healing by second intention.

After such treatments, the second stage of the study also lasted 30 days. At the end of this period, new intraperitoneal anesthesia was performed, followed by euthanasia and resection of a tissue cube approximately 2x2x2 cm, involving the anal canal and the entire fistulous tract, the normal skin around the external orifice, in order to contain the fistulous tract throughout its length, being finally identified and fixed in buffered formol at 10%. 

Subsequently, the material was processed in increasing concentrations of alcohol, diaphanized in xylol, included in histological paraffin, and cross sections of 5 μm thickness were made with the help of a microtome rotator (Microm HM320). The sections obtained were stained by the H&E technique for qualitative histopathological analysis^21^ ^,^ ^22^ and Gomori trichrome (GT) for measurement of the area of muscle separation to evaluate fibrosis sites, giving the green coloration to collagen^2^ ^,^ ^6^.

The capture of the digital images of the slides stained with H&E and GT were performed in a Carl Zeiss photomicroscope coupled to a Samsung micro camera connected to a computer with an image capture card.

**FIGURE 2:**
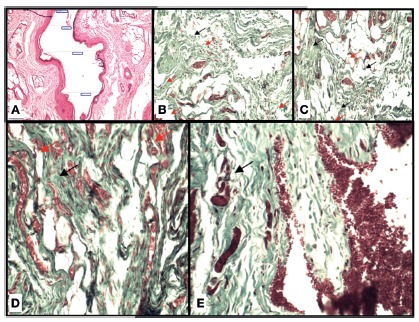
A) Photomicrography illustrating the accomplishment of the measurement of the distance of the muscular cables; B-E) photomicrographies demonstrating the degree of fibrosis: B) control group; C) fistulotomy group; D) polypropylene group; E) polydioxanone group. (Black arrows show collagen fibers and red arrows neoformation of blood vessels, GT, 20x).

The purpose of the histological study was to demonstrate the persistence or healing of the fistulous tracts, using the following criteria: 1) persistence/closure of the fistulous tract - microscopic observation of the fistula persistence was considered to be closed only when the entire tract was closed and the maintenance even if the short length of the tract was considered persistence; 2) distance between the muscular cables made with light microscopy (Figure 2A) in cross section of the anal canal, having measured the perimeter in micrometers (μm) the area in micrometers (μm^2^) between the edges of the muscular cables (fistulotomy group), as well as those sectioned and approximated with primary suture (polydioxanone and polypropylene groups measured by the BioEstat 5.3 program); 3) fibrosis, determined by grade using a scoring system according to the presence of thick collagen fibers and fibroblasts in the tissue in the first stage of granulation, light fibrosis with up to 25% cellularity in the analyzed area, moderate fibrosis with cellularity between 25-50% in the analyzed area, and intense fibrosis with cellularity above 50% in the analyzed area (Figure 2B-E). 

### Statistical analysis

The comparison between the experimental groups, in relation to the area and perimeter of the anal fistula of the animals, was performed using the one-way ANOVA test, followed by the Tukey post-test. The other results of this study were presented in the form of descriptive statistics or in the form of table and graphs. Statistical analysis was performed using the statistical program SigmaPlot, version 12.5, considering a level of significance of 5%^17^.

## RESULTS

At the end of the 30 days, all five animals of the control group, submitted only to the withdrawal of the steel wire, in the histological evaluation showed the lumen circumscribed for granulation tissue, with persistence of the fistula. All animals in the fistulotomy, polydioxanone and polypropylene groups had complete closure of the fistulous tract (Table 1).

**TABLE 1:** Persistence of anal fistula in the animals studied

Animal	Experimental Group			
	Control	Fistulotomy	Polydioxanone	Polypropylene
1	Yes	No	No	No
2	Yes	No	No	No
3	Yes	No	No	No
4	Yes	No	No	No
5	Yes	No	No	No
6	-	-	No	No
7	-	-	No	No
8	-	-	No	No
9	-	-	No	No
10	-	-	No	No
%	100.0	0.0	0.0	0.0
Number	10	0	0	0

The results are presented in their individual values or relative frequency (%) and absolute frequency (number).

Table 2 presents the results concerning to the area of the fistula, its perimeter and the degree of fibrosis caused by the anal fistula, in the animals of the control group.

**TABLE 2:** Results regarding the area, perimeter and degree of fibrosis caused by anal fistula in the animals of the control group.

Animal	Control group		
	Area of anal fistula^*^	Perimeter of anal fistula ^*^	Grade of fibrosis^**^
1	8836	567	1
2	11405	595	2
3	37730	1111	2
4	16931	1195	2
5	29067	1407	1
Mean	20793.80	975.00	1.60
SEM	5483.34	167.99	0.24

The results are presented in their individual values or in mean ± standard error of the mean (SEM); * =in micrometers; ** =fibrosis score

For the analysis of the distance of the muscular cables, there was no difference between the experimental groups. The average distance of the muscular cables in the fistulotomy group was 1620 μm, 4484.80 μm in the polydioxanone group and 4665.50 μm in the polypropylene group (one-way ANOVA test, p=0.078, Table 3).

**TABLE 3:** Results related to the area of distance of the muscle cables in animals from each of the experimental groups.

Animal	Experimental group			p
	Fistulotomy	Polydioxanone	Polypropylene	
1	966	7308	7638	
2	1007	5663	2259	
3	2020	2959	1136	
4	2134	6177	794	
5	1973	2035	385	
6	-	1780	2388	
7	-	5954	8269	
8	-	5402	8501	
9	-	7657	6783	
10	-	3913	8502	
Mean	1620.00^a^	4884.80a	4665.50a	0.078
SEM	260.03	663.81	1117.94	

The results are presented in their individual values or in mean±standard error of the mean (SEM); value of p in the one-way ANOVA test; similar letters in the row indicate that there is no significant difference between the experimental groups (Tukey post-test p>0.05); values presented in micrometers.

According to Table 4, there was also no difference in the degree of fibrosis caused by the different treatments. A mean of 2.3 in the fistulotomy group, 3.1 in the polydioxanone group and 2.8 in the polypropylene group (one-way ANOVA test, p=0.113).

**TABLE 4:** Results regarding the degree of fibrosis caused by the different treatments in the animals of each of the experimental groups.

Animal	Experimental group			p
	Fistulotomy	Polydioxanone	Polypropylene	
1	2	3	3	
2	2	3	2	
3	4	4	2	
4	1	3	1	
5	3	4	4	
6	2	3	3	
7	3	3	3	
8	2	3	4	
9	2	2	4	
10	2	3	2	
Mean	2.30^a^	3.10a	2.80a	0.113
SEM	0.26	0.18	0.33	

The results are presented in their individual values or in mean±standard error of the mean (SEM); value of p in the one-way ANOVA test; similar letters in the row indicate that there is no significant difference between the experimental groups (Tukey post-test, p> 0.05)

## DISCUSSION

The most accepted experimental model of perianal fistulas in the world was initially developed in pigs, since the presence of lumen and visualization of granulation tissue were evidenced by magnetic resonance imaging and in histological analysis of the specimens^3^. The use of rats in the experiment is justified by the fact that the procedure and the care of a smaller animal are easier, in addition to being animals with internal and external sphincter structures similar to human^2^. 

The present study evidenced persistence of the fistulous tract in all the animals of the control group after 30 days of the withdrawal of steel wire with presence of granulation tissue and some epithelization, as already described by Arakaki *et al* ^2^, which observed some degree of epithelialization in 90% of the specimens studied. Important to note that Mitalas *et al* ^16^ in their study evidenced that the epithelization of the fistulous tract does not interfere in the evolution/healing of the patients submitted to advancement of the mucosal flap in the treatment of the anal fistula. The findings of the present study therefore demonstrate that in the model used both fistulotomy and sphincteroplasty, either polydioxanone or polypropylene, they are equally effective in the treatment of anal fistula, since there was no persistent fistulous tract in any of the animals in these groups. However, despite the efficacy of the techniques, it is necessary to evaluate the result in relation to fecal continence, which depends on the sphincter integrity or preservation of the greater part of the sphincter. 

It was believed that the distance between the muscular cables of the animals submitted to sphincteroplasty with polypropylene and polydioxanone would be lower than those submitted only to fistulotomy. However, the groups treated with primary suture with the different suture threads in question did not present any difference in relation to the reduction of the distal area of the muscular cables when compared to each other or to the group that had the curetted fistulous path after the sphincter section. In a randomized clinical trial, the wires 2/0 polyglactin and 3/0 polydioxanone were also compared in the primary repair of sphincter injury after vaginal delivery. After six weeks of treatment, there was no significant difference in the primary outcome of the groups, as well as in the type of wire used in the repair^8^.

When the approach of the muscular cables is promoted, the purpose is to reconstitute the anatomy aiming at a normal or as close to normal sphincter function as possible. It would be pointless to add procedures to the fistulotomy, with longer surgical time, cost and probably more local pain if this did not result in a better functional result. Taking into account the results obtained here is what seems to happen, since there was no anatomical difference in the long term with or without sphincteroplasty after the fistulotomy.

There was no incidence of local abscess in any of the animals submitted to suture treatment, which may be due to the fact that only monofilament yarns were used, corroborating the information that the type of material used in the suture also determines the evolution of the patients. Multifilamentary yarns (polyglactin) predisposes proliferation of microorganisms in a surgical site, delaying the cicatricial process^8^.

It is known that healing of the sphincteric muscle injury begins in the formation of granulation tissue, which is later matured into fibrous tissue and, finally, becomes an infiltrate of muscular fibers from the approximate sphincteric stumps, which may result in recovery from muscle continuity, as well as its function^20^. All groups submitted to the three treatments had the cicatricial process culminating in fibroblast proliferation and collagen deposition, corroborating the information that the primary repair without other associated interventions can achieve acceptable results that can be amplified by the infiltration of stem cells derived of adipose tissue, as previously experienced^20^. 

These findings show that new studies are necessary in order to implement new techniques that stimulate the infiltration/replacement of the fibrous tissue by muscle cells, seeking the perennial morphofunctional reestablishment, with the ultimate objective of quality of life for the patient.

Another important factor to be considered in preserving the sphincter function is the degree of fibrosis. The elasticity of the anal canal is necessary for a complete anus closure under the action of the sphincter contraction, but also for its complete opening when the sphincters are relaxed for satisfactory evacuation. It is very probable, therefore, that the higher the degree of fibrosis in the anal region, the greater the impairment of this function, which, by the results obtained here would place in all the animals in the same condition, since those submitted to fistulotomy only or associated with sphincteroplasty, regardless of the thread used, had the same degree of local fibrosis^2^ ^,^ ^7^.

Clinical studies of patients treated by fistulotomy followed by sphincteroplasty have shown very variable results regarding long-term fecal incontinence, but most of them with acceptable results in the case of trans-sphincteric fistula, below 5% of fecal incontinence^7^ ^,^ ^11^. Such studies have not evaluated the distance of the muscular cables or the degree of fibrosis, but supposing that what is observed in the present research also happens in patients treated with fistulotomy followed by sphincteroplasty, perhaps there is not so much importance in the sphincter continuity and a space between the muscular cables filled by fibrosis could function as a fulcrum for preserving the muscle continence. However, we must consider that clinical research has mostly failed to show a short-term follow-up, and perhaps in old age, there may be incontinence due to the sphincter defect of those patients.

It is important to highlight the safety of the procedures, as there was no significant bleeding in the immediate or late postoperative period, nor the formation of abscesses, although these were not the pourposes of the study. Also the efficacy of the methods in the cure of the fistula, since there was no persistence of fistulous tract in any of the treated animals, in contrast to the control group in which there was persistence of the fistula in all the animals. 

However, it is important to emphasize the need to evaluate in future experiments the functional rehabilitation capacity of the sphincter complex after sphincteroplasty, since the experimental model used here does not allow to evaluate faithfully the sphincter function, only to estimate a probable injury since the muscle cables remained distant in all treated animals.

## CONCLUSIONS

Fistulotomy followed or not by sphincteroplasty was able to eliminate the fistulous tract. The animals treated by sphincteroplasty with polydioxanone or polypropylene yarn presented the same degree of distancing of the muscular cables and fibrosis as those treated only by fistulotomy. 
